# Cholesterol-Dependent *Anaplasma phagocytophilum* Exploits the Low-Density Lipoprotein Uptake Pathway

**DOI:** 10.1371/journal.ppat.1000329

**Published:** 2009-03-13

**Authors:** Qingming Xiong, Mingqun Lin, Yasuko Rikihisa

**Affiliations:** Department of Veterinary Biosciences, The Ohio State University, Columbus, Ohio, United States of America; Medical College of Wisconsin, United States of America

## Abstract

In eukaryotes, intracellular cholesterol homeostasis and trafficking are tightly regulated. Certain bacteria, such as *Anaplasma phagocytophilum*, also require cholesterol; it is unknown, however, how this cholesterol-dependent obligatory intracellular bacterium of granulocytes interacts with the host cell cholesterol regulatory pathway to acquire cholesterol. Here, we report that total host cell cholesterol increased >2-fold during *A. phagocytophilum* infection in a human promyelocytic leukemia cell line. Cellular free cholesterol was enriched in *A. phagocytophilum* inclusions as detected by filipin staining. We determined that *A. phagocytophilum* requires cholesterol derived from low-density lipoprotein (LDL), because its replication was significantly inhibited by depleting the growth medium of cholesterol-containing lipoproteins, by blocking LDL uptake with a monoclonal antibody against LDL receptor (LDLR), or by treating the host cells with inhibitors that block LDL-derived cholesterol egress from late endosomes or lysosomes. However, de novo cholesterol biosynthesis is not required, since inhibition of the biosynthesis pathway did not inhibit *A. phagocytophilum* infection. The uptake of fluorescence-labeled LDL was enhanced in infected cells, and LDLR expression was up-regulated at both the mRNA and protein levels. *A. phagocytophilum* infection stabilized LDLR mRNA through the 3′ UTR region, but not through activation of the sterol regulatory element binding proteins. Extracellular signal–regulated kinase (ERK) was up-regulated by *A. phagocytophilum* infection, and inhibition of its upstream kinase, MEK, by a specific inhibitor or siRNA knockdown, reduced *A. phagocytophilum* infection. Up-regulation of LDLR mRNA by *A. phagocytophilum* was also inhibited by the MEK inhibitor; however, it was unclear whether ERK activation is required for LDLR mRNA up-regulation by *A. phagocytophilum*. These data reveal that *A. phagocytophilum* exploits the host LDL uptake pathway and LDLR mRNA regulatory system to accumulate cholesterol in inclusions to facilitate its replication.

## Author Summary

Maintenance of the cholesterol amount and transport within cells are essential for healthy human cell functions. Most bacteria do not need cholesterol, but certain bacteria that infect human cells are dependent on host cell cholesterol for their infection. How infected human cells deal with these cholesterol-robbing bacteria, and in turn how these bacteria hijack host cholesterol, are intriguing questions. *Anaplasma phagocytophilum* is a bacterium that lives inside white blood cells, and causes the disease human granulocytic anaplasmosis (HGA). *A. phagocytophilum* needs host cholesterol to live. Here, we discovered that *A. phagocytophilum* infection increases the amount of cholesterol in host cells and sequesters the majority of cholesterol in *A. phagocytophilum* inclusions inside host cells. Human cells acquire cholesterol from two sources: receptor-mediated endocytosis of cholesterol-containing low-density lipoprotein (LDL) from the circulating blood, and synthesis of cholesterol inside the cells. Since *A. phagocytophilum* depends on cholesterol derived from LDL, it coaxes the host cell to take up more LDL by increasing LDL receptor, through inhibition of LDL receptor mRNA degradation. *A. phagocytophilum* infection may serve as a model to improve our understanding of the cellular cholesterol regulation in white blood cells, and may provide insight regarding new therapeutic target for treatment of HGA.

## Introduction

Cholesterol is an important component of biological membranes, and it is essential for many biological functions ranging from membrane trafficking to signal transduction in eukaryotic cells [1]. However, excess cholesterol must be avoided in cells as well as in the blood stream, because it alters intracellular vesicular trafficking, deregulates cellular signaling, and initiates atherosclerosis [2],[3]. The liver in large part regulates blood cholesterol levels by removing it from circulating blood. To maintain cellular cholesterol levels within a specified range, cholesterol levels are constantly assessed and tightly regulated in a complex manner at the transcriptional, translational, and posttranslational levels [4]. In recent years, cellular cholesterol has emerged as a significant factor, which influences outcome of infectious diseases from microbiological and cell biological studies. The cholesterol content of host cell membranes appears to be critical for microbial entry, intracellular localization, and exit by exocytosis [5]. A growing body of evidence suggests that host cellular cholesterol levels affect the replication of intracellular microbial pathogens, such as *Salmonella*, *Mycobacterium*, *Brucella*, and *Coxiella* [5],[6],[7], but how cholesterol influences replication of these pathogens are not completely understood. Among the above-mentioned pathogens, infection by *Salmonella* or *Coxiella* up-regulates cellular cholesterol levels, although the mechanisms of up-regulation are not clear [7],[8]. One of the common characteristics for these intracellular bacteria is that after internalization into their host cells the bacteria reside and proliferate in parasitophorous vacuoles. As such, cholesterol may play a role in nutrient acquisition by bacteria entrapped within vacuoles, or the accumulation of cholesterol may prevent phagolysosomal fusion [5].

*Anaplasma phagocytophilum* is a tick-borne obligatory intracellular bacterium that proliferates in membrane-bound inclusions in granulocytes and endothelial cells of various mammal species [9],[10],[11]. In humans, *A. phagocytophilum* causes an emerging and major tick-borne disease called human granulocytic anaplasmosis, an acute febrile disease that is potentially fatal, especially in elderly or immunocompromised individuals [12]. *A. phagocytophilum* is an atypical Gram-negative bacterium, because it contains substantial amounts of cholesterol in its outer membrane [13]. The bacterium lacks genes for cholesterol biosynthesis or modification; rather, it directly acquires cholesterol from its host cells or the medium [13]. Our previous data showed that cholesterol is required for *A. phagocytophilum* proliferation in host human promyelocytic leukemia HL-60 cells and that a high blood cholesterol level facilitates *A. phagocytophilum* infection in a mouse model [13],[14]. *A. phagocytophilum* enters host cells through caveolae or lipid rafts, and the inclusion membrane retains caveolin-1 throughout infection, suggesting continuous infusion of the lipid raft or caveosome into growing bacterial inclusions [15].

Considering cholesterol-dependence of *A. phagocytophilum* membrane integrity and the importance of cholesterol for the infection process, thus survival [13], we questioned how host cellular cholesterol uptake, trafficking, and regulatory systems are involved in *A. phagocytophilum* infection of human leukocytes. In this study, we present data on the intracellular cholesterol level and cholesterol distribution in *A. phagocytophilum*–infected HL-60 cells. We provide evidence that the source of increased level of cellular cholesterol required for *A. phagocytophilum* replication is extracellular low-density lipoprotein (LDL) rather than cholesterol synthesized by the host cells. Finally, we propose a mechanism by which the cellular LDL receptor (LDLR) level is increased in infected HL-60 cells to take up more LDL. The data underscore an important evolutionary adaptation of *A. phagocytophilum* to hijack host cell cholesterol.

## Results

### Total cholesterol level is up-regulated in *A. phagocytophilum*–infected cells

**Figure 1:**
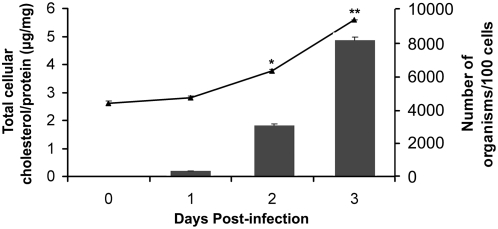
Total cholesterol level is increased in *A. phagocytophilum*–infected HL-60 cells. *A. phagocytophilum*–infected HL-60 cells were harvested on 0, 1, 2 and 3 day p.i., and total cellular cholesterol levels and bacterial burden were determined. Cholesterol concentrations are expressed as micrograms of total cholesterol (esterified and unesterified cholesterol) per milligram of total protein (black triangles). Bacterial burdens are expressed as bacterial numbers per 100 cells (black bars). Data are expressed as mean±standard deviation (n = 3) and are representative of at least three independent experiments with similar results. *, *p*<0.05 (unpaired two-tailed *t* test); **, *p*<0.01 (unpaired two-tailed *t*-test).

We previously measured the total cholesterol level of host cell–free *A. phagocytophilum* and found that the level of total cholesterol per milligram of protein was higher than that of host cells [13]. Here, we further measured the total cholesterol level in *A. phagocytophilum*–infected HL-60 cells following infection time course. The total cellular cholesterol level progressively increased at days 2 and 3 post-infection (p.i.), and the level was significantly greater than that at day 0 p.i. (after 1 h incubation at 37°C). The increase in cholesterol level in infected HL-60 cells correlated with bacterial growth (Figure 1). In contrast, the cholesterol level in uninfected HL-60 cells remained unchanged during the same observation period (data not shown).

### Free cholesterol is enriched in *A. phagocytophilum* inclusions

**Figure 2:**
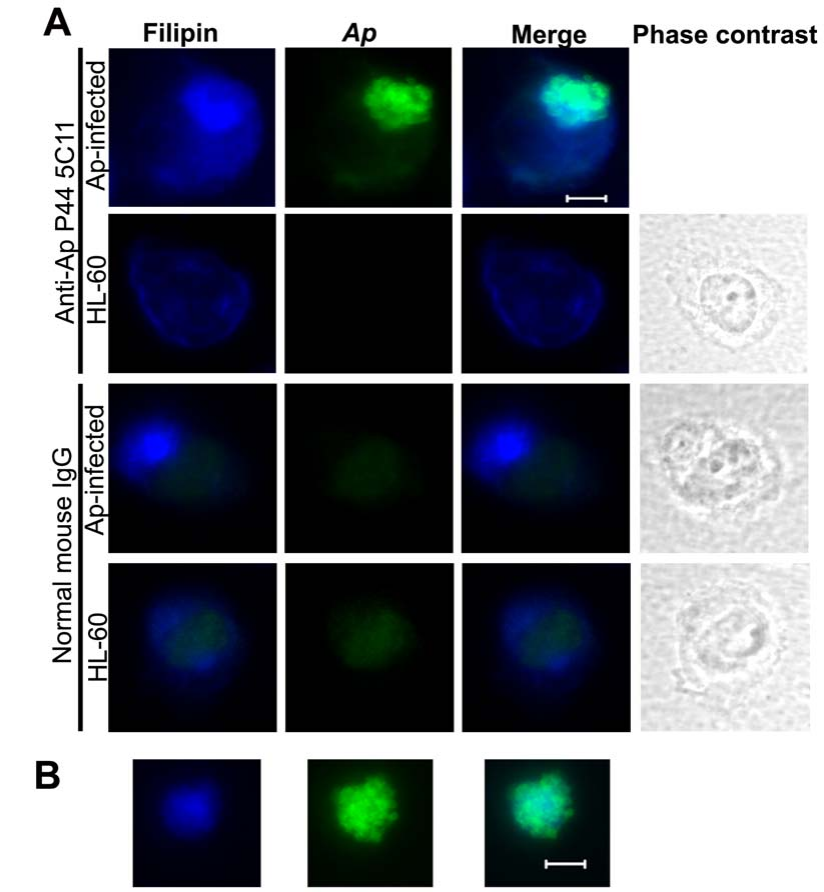
Free cholesterol is enriched in *A. phagocytophilum* inclusions. *A. phagocytophilum*–infected HL-60 cells (A) and *A. phagocytophilum* inclusions released from host cells (B) were fixed at 2 d p.i., stained with mouse anti-*A. phagocytophilum* (green) or normal mouse IgG and filipin (blue), and analyzed by fluorescence microscopy. The experiment shown is representative of at least three independent experiments. Bar, 5 µm. *Ap*, *A. phagocytophilum*.

Most of the free (unesterified) cholesterol in eukaryotic cells is located in the plasma membrane [16]. Over-accumulation of free cholesterol in cells can be toxic due to the potential formation of solid crystals [17]. To determine the intracellular distribution of the observed increased cholesterol in *A. phagocytophilum*–infected HL-60 cells, we used a polyene antibiotic, filipin, which binds specifically to free cholesterol [18]. A specific antibody against *A. phagocytophilum* was used to localize bacteria by double immunofluorescence microscopy. First, the microscopy analysis clearly showed the overall filipin signal was much stronger in *A. phagocytophilum*-infected HL-60 cells than that in uninfected HL-60, which supports the data shown in Figure 1 and further suggests the increased total cellular cholesterol might be free cholesterol, but not esterified cholesterol (Figure 2A). Second, most of the filipin signal was confined in *A. phagocytophilum*–containing vacuoles (“inclusions”) (Figure 2A). Uninfected host cells showed weak filipin signal, which was mostly localized to the plasma membrane and some unknown compartments (assumed to be recycling endocytic compartments [19]). Notably, *A. phagocytophilum* inclusions outside of host cells also clearly displayed strong filipin signals (Figure 2B), suggesting that the inclusion has intrinsic ability to retain the cholesterol. Recently, it was shown that *Chlamydia* release from the infected host cells occurs by two mechanisms: lysis and extrusion [20]. How the *A. phagocytophilum* inclusion became extracellular remains to be studied. Taken together, these results indicate that *A. phagocytophilum* infection alters host intracellular cholesterol homeostasis and distribution and that free cholesterol is enriched in *A. phagocytophilum* inclusions.

### *A. phagocytophilum* infection requires cholesterol derived from the host LDL uptake pathway rather than de novo biosynthesis

Mammalian cells acquire cholesterol from two sources: receptor-mediated uptake from exogenous lipoproteins and endogenous biosynthesis in the smooth ER [4]. In leukocytes, LDL is the primary exogenous cholesterol source that is acquired via LDLR-mediated endocytosis [21]. After hydrolysis of cholesterol esters in acidic late endosomes (enriched in acid lipases), the egress of free cholesterol occurs and free cholesterol is transported to the plasma membrane or delivered to the ER. Excess free cholesterol is catalyzed into cholesteryl esters by the resident ER acyl-CoA: cholesterol acyltransferase and stored as cytoplasmic lipid droplets [2],[22].

**Figure 3:**
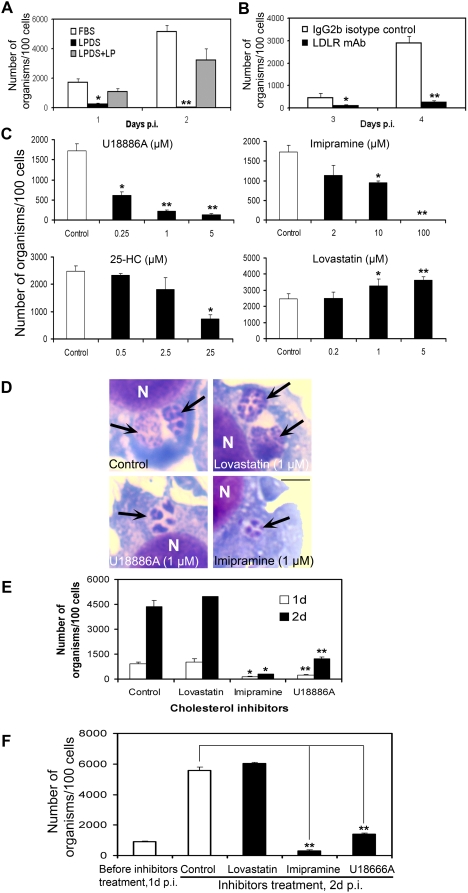
*A. phagocytophilum* infection requires cholesterol derived from the host LDL uptake pathway rather than de novo biosynthesis. (A) *A. phagocytophilum* growth in HL-60 cells cultured in the growth medium containing lipoprotein deficient serum (LPDS), LPDS reconstituted with lipoproteins (LP), and fetal bovine serum were determined on days 1 and 2 p.i. (B) HL-60 cells were pre-treated with anti-LDLR mAb and isotype control mouse IgG and infected with host cell–free *A. phagocytophilum*. The number of bacteria was determined on days 3 and 4 p.i. (C) Cholesterol transport and biosynthesis inhibitors U18886A, imipramine, 25-hydroxycholesterol (25-HC), and lovastatin were added at 1 h p.i. at different dosages, and numbers of bacteria were determined on day 2 p.i. Data are expressed as mean±standard deviation (n = 3) and are representative of two independent experiments with similar results. *, *p*<0.05 (unpaired two-tailed *t* test); **, *p*<0.01 (unpaired two-tailed *t*-test). (D) *A. phagocytophilum*-infected HL-60 cells at 1 h p.i. were treated with lovastatin (1 µM), U18886A (1 µM), imipramine (20 µM), and DMSO vehicle control, respectively. On day 1 p.i., infected cells were harvested and observed by light microscopy following Diff-Quik staining. Arrows indicate *A. phagocytophilum* inclusions. Note much smaller sizes of inclusions that contain much fewer numbers of bacteria in U18886A- or imipramine-treated cells than those in untreated or lovastatin-treated cells. N, nucleus. All figures are shown at the same magnification. Bar, 5 µm. (E) Cultures were treated as in (C), and numbers of bacteria were determined on days 1 and 2 p.i. *, *p*<0.05 (unpaired two-tailed *t* test); **, *p*<0.01 (unpaired two-tailed *t*-test). (F) Lovastatin (1 µM), U18886A (5 µM), imipramine (100 µM), and vehicle control were added to *A. phagocytophilum*-infected HL-60 on day 1 p.i. (∼40% infected cells), and the number of bacteria was determined on day 2 p.i. Data are expressed as mean±standard deviation (n = 3) and are representative of three independent experiments with similar results. *, *p*<0.05 (unpaired two-tailed *t* test); **, *p*<0.01 (unpaired two-tailed *t*-test).

Both undifferentiated and macrophage differentiated HL-60 cells express a regulated LDLR [23]. Cholesterol is essential for *A. phagocytophilum* infection in HL-60 cells [13]; thus, to better understand the source of free cholesterol required for *A. phagocytophilum* infection, we first examined the LDLR-mediated cholesterol uptake pathway using: 1) lipoprotein-deficient serum (LPDS), 2) anti-LDLR monoclonal antibody (mAb), and 3) pharmacological inhibitors of the LDLR-mediated cholesterol uptake pathway. LPDS was prepared from the fetal bovine serum by removing ∼95% lipoproteins using potassium bromide gradient ultracentrifugation (data not shown). The fractionated lipoprotein (LP) was added back to LPDS in certain experiments. LPDS prevented the infection of host cells by *A. phagocytophilum*, and LPDS reconstituted with LP reversed this inhibition (Figure 3A). Moreover, the infection rate of LPDS-conditioned HL-60 cells was decreased on day 2 p.i. compared with that on day 1; and addition of LP rescued the growth on day 2 p.i. (Figure 3A), suggesting that cholesterol derived from LP is essential for *A. phagocytophilum* survival and proliferation in host cells. LDL enters host cells via LDLR-mediated endocytosis, which is blocked by a neutralizing antibody against LDLR [21]. We found that the infection was also significantly blocked by the LDLR mAb (Figure 3B).

U18886A and imipramine are hydrophobic amines that accumulate in acidic cellular compartments, such as lysosomes, and block the post-lysosomal transport of cholesterol in the LDL uptake pathway [24],[25]. We found that both U18886A and imipramine significantly inhibited *A. phagocytophilum* infection and replication in HL-60 cells in a dose-dependent manner. U18886A (5 µM) and imipramine (100 µM) added at 1 h p.i. almost completely blocked bacterial growth (Figure 3C). In contrast, lovastatin, an inhibitor of the rate-limiting enzyme 3-hydroxy-3-methylglutaryl (HMG)-CoA reductase in the cholesterol biosynthetic pathway [26], did not inhibit *A. phagocytophilum* infection, but rather significantly enhanced it in HL-60 cells in a dose-dependent manner (1–5 µM; Figures 3C and D). 25-Hydroxycholesterol (25-HC), which inhibits both LDL uptake and biosynthesis by acting as a negative feedback regulator of cholesterol metabolism [27], partially inhibited *A. phagocytophilum* infection (Figure 3C). Trypan blue and Diff-Quik staining followed by light microscopy showed that although infected host cells remained viable and did not show significant changes, the morphological characteristics of *A. phagocytophilum* inclusions were different: bacterial inclusions remained small in U18886A- or imipramine-treated cells compared with those in untreated cells (Figure 3D). The inhibitory effect of U18886A and imipramine, and the stimulatory effect of lovastatin, were observed at 1 and 2 day p.i. (Figure 3E). When inhibitors were added at 1 day p.i. (40% of HL-60 cells were infected), the bacterial proliferation was still significantly blocked in U18886A- and imipramine-treated cells at optimal concentrations of 5 and 100 µM, respectively (Figure 3F). Taken together, these results indicate that not only *A. phagocytophilum* proliferation, but also survival of the bacterium in inclusions is dependent on cholesterol derived from the LDL uptake pathway, as bacterial numbers declined upon exposure to LPDS or imipramine. Furthermore, de novo cholesterol biosynthesi**s** is not required, and inhibition of this biosynthesis stimulated, rather than inhibited *A. phagocytophilum* infection in HL-60 cells. As *A. phagocytophilum* also infects endothelial cells [10],[11], we used another cell line, monkey endothelial RF/6A to perform the inhibitor studies. Our data showed that the infection of *A. phagocytophilum* in RF/6A was also significantly inhibited by cholesterol transport inhibitors U18666A (5 µM) and imipramine (20 µM). However, no inhibitory effect was observed by lovastatin treatment (1 µM) (Figure S1). Taken together, these data suggest that LDLR-dependent cholesterol uptake pathway is critical for *A. phagocytophilum* infection in both leukocytes and endothelial cells.

### DiI-LDL uptake is enhanced by *A. phagocytophilum* infection in HL-60 cells

**Figure 4:**
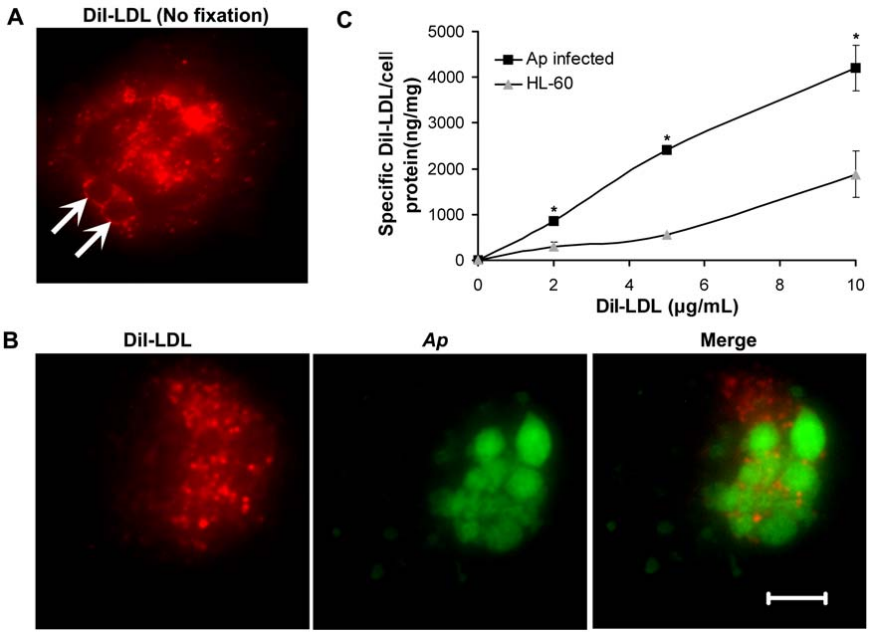
DiI-LDL uptake is enhanced upon *A. phagocytophilum* infection in HL-60 cells. Uninfected and *A. phagocytophilum*–infected HL-60 cells were incubated with increasing concentrations of DiI-LDL at 37°C for 2 h. After DiI-LDL uptake, HL-60 cells were directly observed by fluorescence microscopy without fixation (A), or after being fixed and labeled with anti–*A. phagocytophilum* (B). Arrows indicate *A. phagocytophilum* inclusions. *Ap*, *A. phagocytophilum*. The amount of cell-associated DiI was determined by fluorometric assay. Data were normalized to total cellular protein, and specific association was determined by subtracting the fluorescence measured in the presence of a 30-fold excess concentration of unlabeled LDL from normalized cell-associated DiI fluorescence (C). Data are expressed as mean±standard deviation (n = 3) and are representative of two independent experiments. *, *p*<0.05 (unpaired two-tailed *t* test); **, *p*<0.01 (unpaired two-tailed *t*-test).

Our data have shown that total cellular free cholesterol is increased in *A. phagocytophilum*–infected cells and that the LDL uptake pathway is required for *A. phagocytophilum* infection. We thus used LDL labeled with the fluorescent probe, 1, 1′-dioctadecyl-3, 3, 3′, 3′-tetramethyl indocarbocyanine (DiI–LDL) [28], to compare the overall LDL uptake by *A. phagocytophilum*–infected and uninfected HL-60 cells. We found that LDL uptake was enhanced in *A. phagocytophilum*–infected cells (Figure 4C). After dissociation from LDLR in early sorting endosomes, LDL is directed to late endosomes for hydrolysis of cholesterol esters [2],[22]. By fluorescence microscopy, *A. phagocytophilum* inclusions were surrounded by DiI-LDL-containing small vesicles (Figures 4A and B), in agreement with our previous reports that lysosomes accumulate around *A. phagocytophilum* inclusions [29].

### LDLR mRNA and protein levels are elevated in *A. phagocytophilum*–infected HL-60 cells

**Figure 5:**
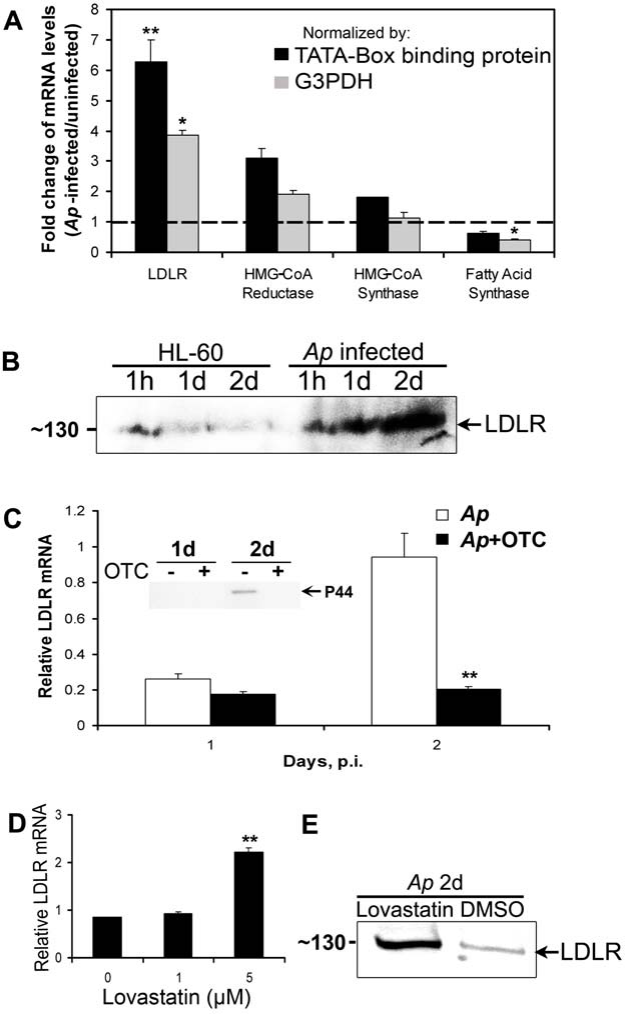
LDLR is up-regulated in *A. phagocytophilum*–infected HL-60 at transcriptional and protein levels. (A) At day 2 p.i., total RNA was extracted from uninfected and *A. phagocytophilum*–infected HL-60 cells. Analysis of mRNA amount was performed using quantitative RT-PCR with specific primers for each gene. Transcript levels were normalized to mRNA level of TATA-box binding protein or G3PDH in each sample. *Ap*, *A. phagocytophilum*. (B) Cell samples were collected at the indicated time points, membrane fractions were isolated, and western blotting was performed to determine LDLR protein levels in uninfected HL-60 and *A. phagocytophilum*–infected HL-60 cells. (C) Oxytetracycline (OTC, 10 µg/ml) was added to *A. phagocytophilum*–infected HL-60 cells at 1 h p.i., RNA was extracted from infected HL-60 cells on days 1 and 2 p.i., and LDLR mRNA was analyzed by quantitative RT-PCR. Transcript levels were normalized to mRNA level of TATA-box binding protein in each sample. Inset shows western blotting result using mAb 5C11 against *A. phagocytophilum* major surface protein P44. (D) Relative LDLR mRNA in lovastatin-treated cell cultures on day 2 p.i. was analyzed by quantitative RT-PCR with primers specific for LDLR gene. (E) LDLR protein levels in lovastatin- or mock (DMSO)-treated infected cells at day 2 p.i. were analyzed by western blotting using anti-LDLR mAb. Data are expressed as mean±standard deviation (n = 3) and are representative of two independent experiments. *, *p*<0.05 (unpaired two-tailed *t* test); **, *p*<0.01 (unpaired two-tailed *t*-test).

We next asked whether the increased cholesterol level and enhanced LDL uptake of host cells upon *A. phagocytophilum* infection may involve up-regulation of LDLR expression. First, we compared mRNA levels of LDLR and other genes involved in cholesterol and fatty acid biosynthesis, including HMG-CoA reductase, HMG-CoA synthase, and fatty acid synthase [30] in infected and uninfected HL-60 cells by real-time RT-PCR. As shown in Figure 5A, LDLR mRNA level was significantly up-regulated at least 4-fold at day 2 p.i. However, the expression of cholesterol biosynthesis genes did not change significantly. The fatty acid synthase gene was significantly down-regulated upon *A. phagocytophilum* infection (Figure 5A). The pattern of LDLR mRNA normalized to host cell TATA-box binding protein mRNA levels was very similar to that normalized to G3PDH mRNA levels. Second, we examined the LDLR protein level by western blotting. As shown in Figure 5B, LDLR was markedly up-regulated upon *A. phagocytophilum* infection. To evaluate whether new protein synthesis or intracellular proliferation of *A. phagocytophilum* is required for LDLR mRNA up-regulation, 10 µg/ml of oxytetracycline was added to cell cultures at 1 h p.i. This treatment completely blocked bacterial proliferation as confirmed by Diff-Quik staining (data not shown) and western blotting using mAb 5C11 against the *A. phagocytophilum* major surface protein, P44 (Figure 5C inset). LDLR mRNA up-regulation at day 2 p.i. was abolished by oxytetracycline treatment (Figure 5C), suggesting that synthesis of new *A. phagocytophilum* proteins and/or intracellular proliferation is required to induce LDLR mRNA up-regulation in HL-60 cells. Notably, real-time RT-PCR analysis showed that LDLR mRNA levels in *A. phagocytophilum*–infected HL-60 cells were significantly increased by lovastatin treatment in a dose-dependent manner (Figure 5D). Western blotting also showed that the LDLR level was markedly higher in lovastatin-treated HL-60 cells at day 2 p.i. compared to DMSO–treated control cells (Figure 5E), which may explain the enhanced infection level in the lovastatin-treated sample as shown in Figure 3. Taken together, these results show that *A. phagocytophilum* infection enhances LDL uptake by up-regulating LDLR expression.

### SREBP cleavage is not involved in the up-regulation of LDLR mRNA in *A. phagocytophilum*–infected HL-60 cells

**Figure 6:**
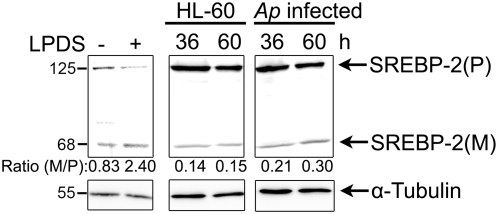
SREBP activation is not up-regulated in *Anaplasma phagocytophilum*–infected HL-60 cells. Uninfected and *A. phagocytophilum*–infected HL-60 cells were collected at the indicated time points, and western blotting was performed using anti-SREBP-2 mAb. α-Tubulin was used as the protein input control to normalize each sample. Positive control was set up by incubating uninfected cells with LPDS-conditioned medium overnight. Ratio of band intensities of mature form to precursor form of SREBP was calculated to show the SREBP-2 cleavage. Data are representative of three independent experiments. *Ap*, *A. phagocytophilum*. P, precursor form of SREBP; M, mature form of SREBP.

Sterol regulatory element binding proteins (SREBPs) are key transcription factors for the cholesterol-mediated feedback regulation to maintain intracellular cholesterol homeostasis by regulating the LDLR gene as well as many cholesterol biosynthesis genes [3]. Three SREBP isoforms have been characterized, namely SREBP-1a, SREBP-1c and SREBP-2 [31]. SREBP-1c, the predominant isoform in adult liver, preferentially activates genes required for fatty acid synthesis, whereas SREBP-2 preferentially activates the LDLR gene and various genes required for cholesterol synthesis, such as HMG-CoA reductase [32]. Therefore, we investigated whether the up-regulation of LDLR by *A. phagocytophilum* infection was due to activation of SREBP-2. SREBPs are activated by cleavage and translocation of the cleaved product from the cytoplasm to the nucleus [3]. As shown in Figures 6, mature cleaved SREBP-2 levels remained unchanged throughout *A. phagocytophilum* infection. As a positive control for SREBP-2 cleavage, uninfected cells were incubated with LPDS-conditioned medium overnight, as this is known to induce SREBP-2 cleavage in different cell lines including CHO and human monoblastic leukemia cell line U937 [33]. There was no significant difference between *A. phagocytophilum*–infected and uninfected HL-60 cells at any post-infection time point examined, suggesting that SREBP-2 activation is not involved in the up-regulation of LDLR mRNA upon *A. phagocytophilum* infection in HL-60 cells. In another word, the dramatic intracellular cholesterol homeostasis up-shift by *A. phagocytophilum* infection cannot be sensed by the host cell key regulatory factor SREBP-2.

### Stabilization of the LDLR mRNA during *A. phagocytophilum*–induced LDLR mRNA up-regulation

**Figure 7:**
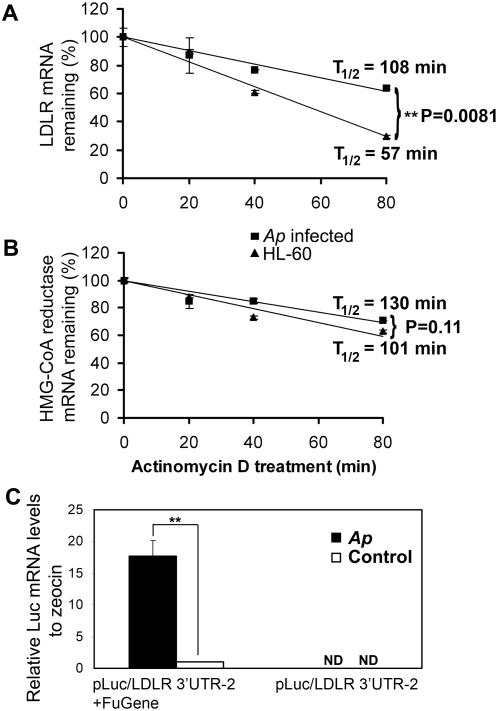
LDLR mRNA is stabilized in *A. phagocytophilum*–infected host cells. Actinomycin D (5 µg/ml) was added to uninfected and *A. phagocytophilum*–infected HL-60 cells for different periods. Total RNA was isolated, and LDLR mRNA (A) and HMG-CoA reductase mRNA (B) were analyzed by quantitative RT-PCR. Data are expressed as mean±standard deviation (n = 3) and are representative of two independent experiments. The decay rates of LDLR mRNA (A) are significantly different (*p*<0.01), as tested by two-way ANOVA. (C) Chimeric pLuc/LDLR 3′UTR-2 was transfected into RF/6A cells using FuGene HD reagent, and host cell–free *A. phagocytophilum* was added to transfected RF/6A cells at 1 day post transfection. Luc mRNA was analyzed on day 2 p.i. by quantitative RT-PCR. Cell samples were normalized by the antibiotic gene zeocin mRNA level. Data are representative of two independent experiments. *Ap*, *A. phagocytophilum*. ND, not detectable. **, *p*<0.01 (unpaired two-tailed *t*-test).

LDLR is regulated not only at the transcriptional level but also at the posttranscriptional level via modulation of LDLR mRNA stability [34]. Therefore, we investigated LDLR mRNA stability in *A. phagocytophilum*–infected HL-60 cells after treatment with actinomycin D, a eukaryotic DNA-dependent RNA polymerase inhibitor. The half-life of LDLR mRNA in infected HL-60 cells was increased by almost 2-fold as compared to uninfected cells (Figure 7A). In contrast, the stability of HMG-CoA reductase mRNA did not change noticeably during *A. phagocytophilum* infection (Figure 7B). Human LDLR mRNA contains a 2.5-kb 3′UTR [35]. The 3′UTR of LDLR mRNA can be stabilized by phorbol-12-myristate-13-acetate (PMA) and a Chinese herbal compound, berberine in the human hepatic cell line HepG2 [36],[37]. Three AU-rich elements (AREs) are located in the 5′ proximal region of the 3′UTR, which have been shown to be responsible for the stabilization of LDLR mRNA by berberine, but not by PMA [37]. To investigate whether the LDLR 3′UTR containing three AREs is involved in *A. phagocytophilum*–induced LDLR stabilization, we transfected the luciferase fusion plasmid pLuc/LDLR 3′UTR-2, containing three AREs of LDLR 3′UTR (nt 2,677–3,582) [37], into RF/6A cells and measured the luciferase mRNA levels in *A. phagocytophilum*–infected and control RF/6A cells. As shown in Figure 7C, luciferase mRNA levels normalized to the antibiotic zeocin resistance gene (plasmid copy number) were significantly increased in *A. phagocytophilum*–infected cells. Data normalized by G3PDH (host cell number) showed a similar pattern (data not shown). These results indicate that the LDLR 3′UTR containing three AREs may be involved in enhancing LDLR mRNA stability in *A. phagocytophilum*–infected host cells.

### ERK activation which is known to up-regulate LDLR expression, is required for *A. phagocytophilum* infection

**Figure 8:**
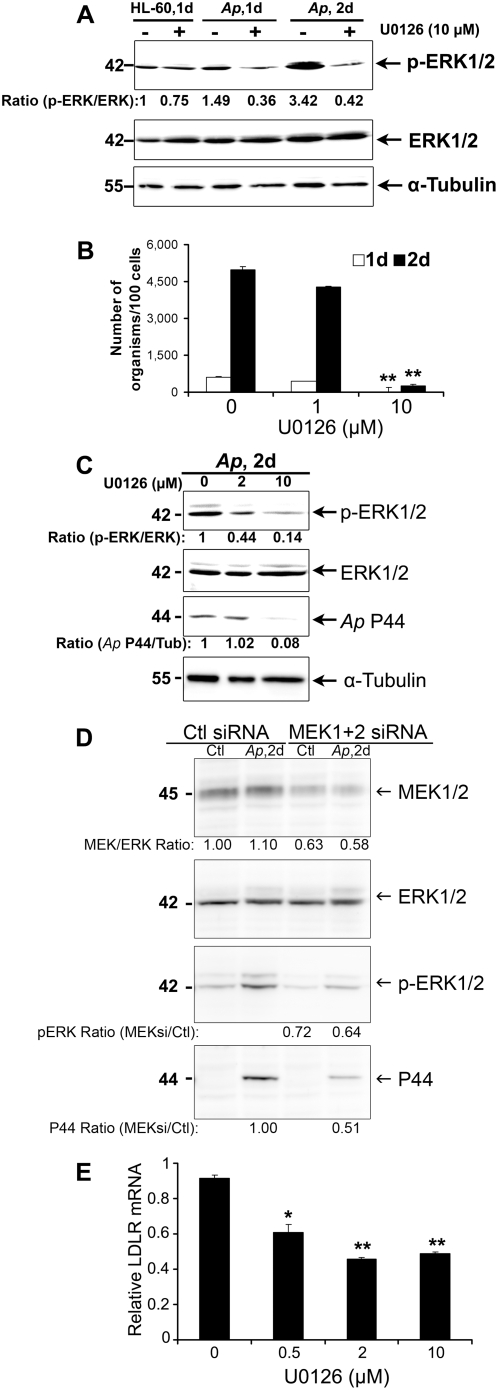
ERK signaling pathway is involved in the LDLR up-regulation upon *A. phagocytophilum* infection. (A) Western blot analysis was performed using antibodies specific to either the phosphorylated or total ERK1/2. Uninfected and *A. phagocytophilum*–infected HL-60 cells with or without 10 µM ERK inhibitor U0126 treatment were collected at the indicated time points. α-Tubulin was used as the protein loading control to normalize each sample. The values under the bands show the ratios of band intensities of p-ERK and ERK. *Ap*, *A. phagocytophilum*. (B) Bacterial numbers per 100 cells treated with the indicated concentrations of U0126 were determined on days 1 and 2 p.i. (C) Western blot analysis of *A. phagocytophilum*–infected HL-60 cells on day 2 p.i. treated with the indicated concentrations of U0126 was performed using antibodies specific to phosphorylated or total ERK1/2, or *A. phagocytophilum* outer membrane protein P44. α-Tubulin was used as the protein input control to normalize each sample. The values under the bands show the ratios of band intensities of p-ERK and P44, normalized to total ERK or α-tubulin, respectively. Data are representative of three independent experiments. (D) HL-60 cells were transfected with control (Ctl) or double-stranded siRNA specific targeting the genes encoding MEK1 and MEK2 (MEK1+2) (3 µg/2×10^6^ cells) using the Amaxa Nucleofection system. Two days after transfection, host cell-free *A. phagocytophilum* was added to the cells and incubated for additional 2 days. One aliquot of samples were lysed and subjected to Western blotting using antibodies against MEK1/2, ERK1/2, phospho-ERK1/2, and *A. phagocytophilum* P44 protein. The relative protein amount of MEK1/2, phospho-ERK1/2, and P44 were determined using total ERK1/2 as loading control by densitometry analysis. The values under the bands show the relative ratios of band intensities, with the ratios of those from samples nucleofected with control siRNA arbitrarily set as 1. Results are representative of three independent experiments. (E) LDLR expression in *A. phagocytophilum*–infected HL-60 cells on day 2 p.i. treated with the indicated concentrations of U0126 was determined by quantitative RT-PCR. Data are expressed as mean±standard deviation (n = 3) and are representative of two independent experiments. *, *p*<0.05 (unpaired two-tailed *t* test); **, *p*<0.01 (unpaired two-tailed *t*-test).

Accumulating evidence suggests that the extracellular signal–regulated kinase (ERK) signaling cascade regulates the induction of LDLR expression in HepG2 cells [34],[38]. Moreover, berberine increases LDLR expression at the posttranscriptional level via ERK-dependent stabilization of LDLR mRNA [37]. Therefore, we asked whether and when ERK1/2 (p44/p42) is activated during *A. phagocytophilum* infection, whether ERK1/2 activation is required for *A. phagocytophilum* infection, and whether ERK1/2 activation up-regulates LDLR, constituting a positive feedback loop for *A. phagocytophilum* replication. First, we determined whether ERK1/2 is activated by *A. phagocytophilum* infection in HL-60 cells by western blotting using an antibody that only recognizes activated (phosphorylated) ERK1/2 but not inactive ERK1/2, as well as an antibody that recognizes both unphosphorylated and phosphorylated forms of ERK. We found that *A. phagocytophilum* activated ERK signaling especially during the exponential growth stage (day 2 p.i.) compared to day 1 p.i. or uninfected HL-60 cells (Figure 8A). Second, we examined the effect of U0126, the inhibitor of ERK upstream kinase MEK1/2, on ERK activation upon *A. phagocytophilum* infection. ERK activation by *A. phagocytophilum* infection and *A. phagocytophilum* replication in HL-60 cells were also almost completely inhibited by 10 µM U0126 (Figures 8A and B). This result was confirmed by western blotting for *A. phagocytophilum* P44 (Figure 8C). To confirm ERK activation is required for *A. phagocytophilum* infection, MEK1/2 proteins were knocked down by RNA interference with siRNAs targeting the MEK1 and MEK2 genes. Results showed that at 4 days post transfection, the protein amount of MEK1/2 was reduced by ∼40% in MEK1/2 knockdown group, which resulted in the partial inhibition of the phosphorylation of ERK1/2 (∼30%), as well as the infection of *A. phagocytophilum* (∼50%)(Figure 8D). These data clearly demonstrate that ERK signaling is activated by and required for *A. phagocytophilum* infection in HL-60 cells.

To determine whether ERK activation is involved in up-regulation of LDLR upon *A. phagocytophilum* infection, we examined the LDLR mRNA levels in U0126-pretreated HL-60 cells by real-time RT-PCR. As shown in Figure 8E, the relative LDLR mRNA level upregulated by *A. phagocytophilum*–infected HL-60 cells was significantly reduced by U0126 treatment starting at a concentration of 0.5 µM at 2 day p.i. Although the expression of LDLR mRNA was slightly reduced in uninfected HL-60 cells by MEK1/2 siRNA knockdown, there was no significant reduction of LDLR mRNA in infected HL-60 cells (data not shown). Therefore, we could not draw the definitive conclusion whether MEK1/2→ERK1/2 pathway is required for up-regulation of LDLR expression in the case of *A. phagocytophilum* infection of HL-60 cells.

## Discussion

In this study, we present evidence that *A. phagocytophilum* is dependent on cholesterol derived from the LDLR-mediated uptake pathway of eukaryotic host cells. Several vacuole-occupying intracellular pathogens depend on host cholesterol stores or trafficking during their infection of cultured cells or mice [5], although mechanisms vary considerably. *Salmonella enterica* serovar Typhimurium requires nonsterol precursors of the cholesterol biosynthetic pathway for intracellular proliferation [39], and cholesterol accumulates in *Salmonella*-containing vacuoles in a *Salmonella* pathogenecity island-2–dependent manner [8],[40]. The establishment of *Brucella abortus* infection in mice requires trafficking of plasma membrane cholesterol, which is controlled by Niemann-Pick C1, an important cholesterol transport protein in late endosomes/lysosomes, as evidenced by resistance to *B. abortus* infection in Niemann-Pick C1 knockout mice [6]. *Chlamydia trachomatis* inclusions acquire cholesterol by selectively rerouting Golgi-derived vesicles [41] and multivesicular bodies [42]. Interestingly, cytoplasmic lipid droplets are translocated into the lumen of *Chlamydia* inclusions, which appears to be an alternate mechanism for acquisition of cholesterol [43]. *Coxiella burnetii* infection increases production of host cell cholesterol with concomitant up-regulation of host genes involved in cholesterol metabolism, including LDLR and several cholesterol biosynthesis genes [7]. Unlike any of the above described intracellular pathogens, *A. phagocytophilum* acquires cholesterol preferentially from the LDL uptake pathway by up-regulating LDLR expression. *Toxoplasma gondii*, although a eukaryotic pathogen, cannot synthesize sterols via the mevalonate pathway and appropriates cholesterol to its parasitophorous vacuole exclusively from LDL uptake [44]. Recently, an unexpected novel mechanism was demonstrated: *T. gondii* actively sequesters the host endocytic vesicles in its vacuolar spaces to provide its cholesterol needs [45]. Interestingly, *A. phagocytophilum* has an intracellular compartment somewhat similar to that of *T. gondii* [29],[46], which is segregated from both endocytic and exocytic pathways.

The present study raises an intriguing question: how does *A. phagocytophilum* acquire cholesterol derived from the host LDL endocytic pathway? Although *A. phagocytophilum* inclusions do not fuse with lysosomes, our previous data clearly demonstrate that these inclusions are surrounded by host lysosomes [29]. The physical proximity between the *A. phagocytophilum* inclusions and host lysosomes may facilitate cholesterol acquisition by *A. phagocytophilum*. Not only *A. phagocytophilum* enters host cells via caveolae-containing lipid rafts, but also the caveolar marker protein, caveolin-1, co-localizes with both early and replicative *A. phagocytophilum* inclusions [13]. Caveolin-1 is a well-established cholesterol binding protein [47], and caveolae/caveosomes have been proposed to be a cholesterol transporter participating in the bidirectional shuttling of free cholesterol between the plasma membrane and various intracellular compartments, including the ER, Golgi and lipid droplets [48]. Therefore, we hypothesize that the caveosome is involved in cholesterol transport to *A. phagocytophilum* inclusions. Another finding showed that some *A. phagocytophilum* inclusions co-localize with vesicle-associated membrane protein 2 (VAMP2) [29]. In neutrophils, VAMP2 is believed to play a role in controlling vesicular targeting, docking, and fusion through interactions with other proteins, such as *N*-ethylmaleimide-sensitive factor and soluble *N*-ethylmaleimide-sensitive factor attachment protein [49]. The presence of VAMP2 on *A. phagocytophilum* inclusions may provide a mechanism to acquire cholesterol for the replicating organism through regulated vesicle trafficking. A recent report showed that *A. phagocytophilum* modulates lipid metabolism by increasing perilipin mRNA and protein levels to facilitate infection of HL-60 cells [50]. Perilipin is a major adipocyte lipid droplet–associated protein that plays a central role in lipolysis and cholesterol synthesis. It is unknown whether lipid droplets serve as an intermediate organelle for cholesterol acquisition by *A. phagocytophilum* after intracellular delivery via the LDLR pathway. Further work is required to determine the exact mechanism of cholesterol acquisition by *A. phagocytophilum*.

Our data demonstrate that *A. phagocytophilum* up-regulates LDLR expression in HL-60 cells by stabilizing the LDLR mRNA via a posttranscriptional mechanism. This is the first report on modulation of LDLR mRNA stability by an infectious agent, and to our knowledge this is also the first report regarding LDLR up-regulation on leukocytes. This aspect may have clinical relevance because increasing hepatic LDLR expression is currently one of the primary strategies for hypercholesterolemia therapy. Recent data suggest that mRNA stability is the major mode of posttranscriptional regulation of LDLR expression. The stability of LDLR mRNA is known to be modulated by only a few reagents, including gemfibrozil [51], PMA, chenodeoxycholic acid and berberine [34]. Interestingly, the stabilization of LDLR mRNA via the 3′UTR by chenodeoxycholic acid (CDCA) and berberine requires activation of the ERK signaling pathway. However, it is so far unclear how the ERK pathway is linked to LDLR mRNA stabilization and whether any trans-acting RNA binding proteins are involved in the stabilization process. In addition, the activity of berberine to up-regulate LDLR expression is specific to hepatocytes, as the significant increase of LDLR was found only in HepG2 cells, but not in other non-hepatic cell lines, such as CHO, HEK293, or human primary fibroblasts [52]. The present study shows that *A. phagocytophilum* potently activates the ERK signaling pathway, especially at the exponential growth stage when the bacterium requires substantial amounts of cholesterol for proliferation with concomitant expansion of inclusions. The result is somewhat consistent with the recent report that *A. phagocytophilum* activates ERK2 (p42) in host human neutrophils at 3 h p.i. [53]. For the first time, our MEK inhibitor and siRNA knock-down studies showed ERK activation is required for *A. phagocytophilum* infection. It has been reported that several other intracellular bacteria actively manipulate the host ERK signaling pathway to benefit microbial survival. Tapinos and Rambukkana reported a PKCε-dependent, but not MEK-dependent pathway for ERK1/2 activation by *Mycobacterium leprae* resulting in continuous proliferation of infected human Schwann cells, without inducing transformation [54]. Interestingly, activation of the host Ras-Raf-MEK-ERK-cPLA2 signaling cascade is required for chlamydial acquisition of host glycerophospholipids [55]. It remains to be elucidated why ERK activation is required for *A. phagocytophilum* infection or whether ERK involves in LDLR mRNA stabilization.

Recently, the use of cholesterol biosynthesis inhibitors, such as statins, was proposed to combat certain pathogen infections because these microbes utilize the host cholesterol biosynthesis pathway. For example, lovastatin and atorvastatin reduce *S. enterica* serovar Typhimurium proliferation in vitro and in BABL/C mice, respectively [39]. Growth of *C. burnetii* can be also inhibited by lovastatin in vitro [7]. It is important to point out that unlike *Salmonella* and *Coxiella*, lovastatin enhanced, at least, did not reduce *A. phagocytophilum* infection in both HL-60 promyelocytic and RF/6A endothelial cells. This result is not surprising, however, because our present data show that *A. phagocytophilum* acquires cholesterol derived from the LDL uptake pathway and not from the biosynthesis pathway. Additionally, lovastatin-treated *A. phagocytophilum*–infected HL-60 cells expressed higher levels of LDLR. Thus, the question then becomes: is statin treatment beneficial, if not detrimental for *A. phagocytophilum* infection in vivo? The answer to this question is very important because statin drugs are widely used in elderly patients to treat hypercholesterolemia, and *A. phagocytophilum* infection is more prevalent in this community. Our recent data showed that high blood cholesterol facilitates *A. phagocytophilum* infection in a mouse model [14]. However, our unpublished data suggest that there is a higher *A. phagocytophilum* level in the blood after Lipitor (atorvastatin) treatment of mice (Xueqi Wang and Yasuko Rikihisa, unpublished data). We had originally hypothesized that statin treatment lowers blood cholesterol levels and consequently results in lower bacterial burden in the mice. This paradox could be explained as follows: statins do not reduce overall plasma cholesterol levels in mice, as they do in humans, due to very low levels of LDLs in rodents, even though they do block mouse HMG-CoA reductase and the sterol biosynthetic pathway in mice [56]. In fact, we have not found decreased blood cholesterol levels in statin-treated mice (Xueqi Wang and Yasuko Rikihisa, unpublished data). Similarly, there is no significant change in serum cholesterol levels in atorvastatin-treated mice compared with vehicle-treated mice, although statins reduce *S. enterica* serovar Typhimurium growth in vivo [39]. Therefore, we speculate that, similar to our in vitro HL-60 cell culture model, up-regulation of LDLR in *A. phagocytophilum*–infected host leukocytes may result in a greater bacterial burden in statin-treated mice. Taken together, these results suggest that critical and careful consideration is required when treating granulocytic anaplasmosis patients, as statins are currently widely used to treat hypercholesterolemia in humans by lowering blood cholesterol levels.

The data presented here improve our understanding of how a cholesterol-dependent bacterium exploits eukaryotic cellular cholesterol trafficking and regulatory pathways and may provide insight regarding a new therapeutic target for the treatment of human granulocytic anaplasmosis. Most information on LDLR regulation is derived from studies using hepatocytes [34]; however, as in the berberine's case [52], in non-hepatic cells, LDLR regulation by cholesterol modulating compounds may differ from those of hepatocytes. Our study enhances our understanding of the LDLR regulation pathway in leukocytes and perhaps endothelial cells [10],[11], both of which are understudied, but important players in atherosclerosis.

## Methods

### Chemicals and antibodies

Filipin, lovastatin, imipramine and U18666A were obtained from Sigma (St. Louis, MO). 25-Hydroxycholesterol (25-HC) was purchased from Steraloids, Inc. (Newport, RI). MAPK inhibitor U0126 was obtained from Biomol (Plymouth Meeting, PA). DiI-LDL and native LDL were purchased from Molecular Probes (Eugene, OR) and Intracel, Inc. (Frederick, MD), respectively.

The mouse mAb 5C11 recognizing the N-terminal conserved region of *A. phagocytophilum* major surface protein P44 has been described [57]. The anti-LDLR mAb was purified from the supernatant of hybridoma ATCC CRL-1691 (C7) grown in advanced MEM (ATCC, Manassas, VA) by affinity chromatography using HiTrap Protein G HP (GE Healthcare, Piscataway, NJ) according to the manufacturer's instructions. The purity of the antibody was confirmed by SDS-PAGE followed by GelCode Blue staining (Pierce, Rockford, IL). Other antibodies used include: mouse anti-SREBP-2 mAb (BD Parmingen, San Jose, CA), mouse anti-phospho-ERK1/2 mAb, rabbit anti-ERK1/2 antibody, mouse anti-MEK1/2 mAb (Cell Signaling, Danvers, MA), and mouse anti-α-tubulin mAb (Santa Cruz Biotechnology, Santa Cruz, CA). Peroxidase-conjugated secondary antibodies were obtained from KPL (Gaithersburg, MD). Normal mouse IgG were purchased from Santa Cruz Biotechnology.

### Preparation of LPDS and lipoproteins

LPDS was prepared from fetal bovine serum (Mediatech, Inc., Herndon, VA) by gradient ultracentrifugation after density adjustment by solid KBr as described [58],[59]. LPs were used to supplement LPDS, as necessary for certain experiments. LPDS and LP fractions were dialyzed at least 36 h against buffer containing 0.15 M NaCl and 0.3 mM EDTA, pH 7.4. The volume of each fraction was adjusted to be equivalent to that of the original serum, and the cholesterol concentration of each fraction was measured by Infinity™ cholesterol reagent kit (Thermo Electron Corp., Louisville, CO).

### *A. phagocytophilum* culture and inhibitor treatments

*A. phagocytophilum* HZ strain was cultivated in human promyelocytic leukemia cell line HL-60 as described [60]. Host cell-free *A. phagocytophilum* was prepared by sonicating highly infected (>90% infected cells) HL-60 cells for 8 s twice at an output setting of 2 with an ultrasonic processor (W-380; Heat Systems, Farmington, NY). After low-speed centrifugation to remove nuclei and unbroken cells, the supernatant was centrifuged at 10,000×*g* for 10 min, and the pellet enriched with host cell–free organisms was added to HL-60 or RF/6A cells. After 1 h incubation at 37°C, extracellular organisms were washed, fresh medium was added (this time point was considered 0 h p.i.), and continuously incubated at 37°C.

Inhibitors were added at indicated time points (0 h or 1 day p.i.), and the inhibitors were kept in the growth media throughout the incubation period or removed later as indicated. The highest final concentrations of inhibitors used were: lovastatin (5 µM), imipramine (100 µM), U18666A (5 µM), and 25-HC (25 µM). Inhibitor treatments at these concentrations did not affect host cell integrity as assessed by light microscopy or by G3PDH mRNA level. For LPDS treatment, 10% LPDS or LP-reconstituted LPDS conditioned growth medium was added at 0 h p.i. in place of the growth medium containing 10% fetal bovine serum. To block LDLR function, HL-60 cells were pretreated with anti-LDLR (IgG_2b_; final concentrations:20 µg/ml) or IgG_2b_ isotype control antibody at 4°C for 1 h followed by addition of host cell–free bacteria, and then culture was continued at 37°C for the indicated times.

The degree of bacterial infection in host cells was assessed by Diff-Quik staining (Baxter Scientific Products, Obetz, OH), and the number of *A. phagocytophilum* cells was estimated in 100 host cells in triplicate culture wells as described [61].

### Cholesterol assay of infected cells

Uninfected and *A. phagocytophilum*–infected HL-60 cells at the indicated time points (1 h, 1 day, 2 day and 3 day) were collected, and total cellular cholesterol levels were measured by an Amplex Red cholesterol assay kit (Molecular Probes) as described [13]. The total cholesterol content was normalized by the total protein concentration as determined by bicinchoninic acid reagent (Pierce).

### DiI-LDL uptake assay

DiI-LDL uptake by infected and uninfected HL-60 cells was measured by the modified method of Teupser et al. [62]. Briefly, uninfected and approximately 40% infected HL-60 cells were incubated with LPDS-conditioned medium for 12 h to enhance LDLR expression. Then, increasing concentrations of DiI-LDL (2, 5, 10 µg protein/ml) with or without 30-fold excess of unlabeled LDL were added to HL-60 cells and incubated for 2 h at 37°C. The cells were thoroughly washed with phosphate-buffered saline (PBS, 137 mM NaCl, 2.7 mM KCl, 10 mM Na_2_HPO_4_, and 2 mM KH_2_PO_4_, pH 7.4) containing 0.4% bovine serum albumin, and lysed in the lysis reagent (0.1% SDS/0.1 N NaOH) for 1 h with gentle shaking. Cellular uptake of DiI-LDL was measured in a fraction (200 µl) of the lysate by fluorescence spectroscopy with excitation and emission wavelengths of 520 and 580 nm, respectively. The fluorometric data were normalized by the total protein content.

### Filipin staining and immunofluorescence microscopy

Filipin staining was performed as described by Millard et al [63]. Cells were fixed in 4% paraformaldehyde at room temperature for 15 min and incubated with 50 µg/ml filipin in PBS/10% normal sheep serum for 30 min at room temperature. Then the cells were incubated with mouse anti–*A. phagocytophilum* antiserum in filipin/PBS/10% normal sheep serum for 60 min at 37°C followed by incubation with fluorescence-conjugated secondary antibodies for 30 min. Normal mouse antibodies were used as negative controls. Cells were then washed and observed under a Nikon Eclipse E400 fluorescence microscope with a xenon-mercury light source (Nikon Instruments, Melville, NY).

### Western blot analysis

*A. phagocytophilum*–infected HL-60 and control HL-60 cells (2×10^6^) were washed and resuspended in 100 µl PBS containing freshly added protease inhibitor cocktail set III and phosphatase inhibitor cocktail set II (Calbiochem, San Diego, CA), and lysed by mixing with 100 µl of 2×Laemmli sample buffer (4% SDS, 135 mM Tris-HCl [pH 6.8], 20% glycerol, and 10% β-mercaptoethanol). Samples were separated by SDS-PAGE with 7.5% or 10% polyacrylamide resolving gels and then transferred to a nitrocellulose membrane using a semidry blotter (WEP, Seattle, WA). The membrane was blocked using 5% (wt/vol) skim milk (Kroger, Cincinnati, OH) in Tris-buffered saline (150 mM NaCl and 50 mM Tris at pH 7.5) containing 0.1% Tween-20, incubated with primary antibodies (1∶500 or 1∶1,000 dilution) at 4°C for 12 h, and subsequently incubated with peroxidase-conjugated secondary antibodies at 1∶1,000 dilution at room temperature for 1 h. Immunoreactive bands were visualized with enhanced chemiluminescence. To detect LDLR protein amount, the membrane fraction of cells was prepared according to Holla et al. [64], and Western blotting was carried out as described [65].

### Quantitative RT-PCR

Uninfected and *A. phagocytophilum*–infected HL-60 cells were harvested, and RNA was isolated using the RNeasy kit (Qiagen, Valencia, CA). Total RNA (2 µg) was reverse transcribed using SuperScript III reverse transcriptase (Invitrogen, Carlsbad, CA) and oligo(dT)12–18 primer (Invitrogen). Quantitative PCR (20 µl total volume) was performed with 1 µl of cDNA (corresponding to 0.2–0.4 µg of total RNA) and 0.25 µM of each primer using a SYBR Green PCR kit (Stratagene, La Jolla, CA) in a Mx3000P Real-time PCR system (Stratagene). All primers for cholesterol-related genes were described in Castoreno et al. [30] and G3PDH primers were described in Zhang et al. [66].

### Transfection of RF/6A cells and quantitative RT-PCR analysis of Luc transcript

The chimeric plasmid pLuc/LDLR 3′UTR-2 has been described [37]. The constructs were sequenced, and individual clones were propagated to isolate plasmid DNA using the Endofree plasmid maxi kit (Qiagen). The plasmids were transfected into endothelial cells, RF/6A, using the FuGene transfection reagent (Roche, Indianapolis, IN). After 24 h, host cell–free *A. phagocytophilum* purified from highly infected HL-60 cells were inoculated into transfected RF/6A cells and incubated for additional 24–48 h. Samples were collected, and first-strand cDNA was synthesized as described above following DNase I (Invitrogen) treatment. The Luc transcripts were measured by quantitative real-time RT-PCR using specific primers: forward, TCCAACCCGGTAAGACACGACT, and reverse, TCAGCAGAGCGCAGATACCAAATA. Host cell G3PDH and the plasmid antibiotics gene zeocin (forward, GACGACGTGACCCTGTTCATCAGC; reverse, CACTCGGCGTACAGCTCGTCCAG) were used for normalization.

### RNA interference

HL-60 cells were transfected with double-stranded siRNA (3 µg/2×10^6^ cells) using the Amaxa nucleofection system (kit V, program T-19; Lonza/Amaxa Inc, Walkersville, MD) as described previously [67]. Verified human-specific siRNAs targeting the genes encoding MEK1 (siRNA ID: s11167) and MEK2 (siRNA ID: s11170), or control siRNA (# 4390843) not targeting any known human genes were purchased from Ambion (Applied Biosystems/Ambion, Austin, TX). Two days after transfection, host cell-free *A. phagocytophilum* was added to cells and incubated for additional 2 days. Samples were then harvested and divided into two aliquots. One group of samples was lysed in M-PER lysis buffer (Pierce) supplemented with protease and phosphatase inhibitor cocktail (Calbiochem), and subjected to Western blotting using antibodies against MEK1/2, ERK1/2, phospho-ERK1/2 and *A. phagocytophilum* P44 outer membrane protein [58]. Images were then captured and densitometric analysis was performed using LAS3000 image documentation system (FUJIFILM Medical Systems USA, Stamford, CT). The other aliquots were stored in RNALater for further quantitative real-time RT-PCR analysis as described above.

### Statistical analysis

Statistical analyses were performed by unpaired, 2-tailed Student's *t*-test. Two-way ANOVA was used to compare mRNA decay rates. *p*<0.05 was considered to be significant.

## Supporting Information

Figure S1 Cholesterol transport and biosynthesis inhibitors U18886A, imipramine, and lovastatin were added into RF/6A cells at 3 h p.i. at the indicated dosages, and infection percentage (A) and numbers of bacteria (B) were determined on day 3 p.i.. Data are expressed as mean±standard deviation (n = 3) and are representative of three independent experiments with similar results. **, *p*<0.01 (unpaired two-tailed *t*-test).
(0.04 MB PDF)
