# Supplementary figures and images for: Cholesterol-Dependent Anaplasma phagocytophilum Exploits the Low-Density Lipoprotein Uptake Pathway
Source: PLoS Pathog. 2009 Mar 13;5(3):e1000329. doi: 10.1371/journal.ppat.1000329 (PMC2654415; doi:10.1371/journal.ppat.1000329)

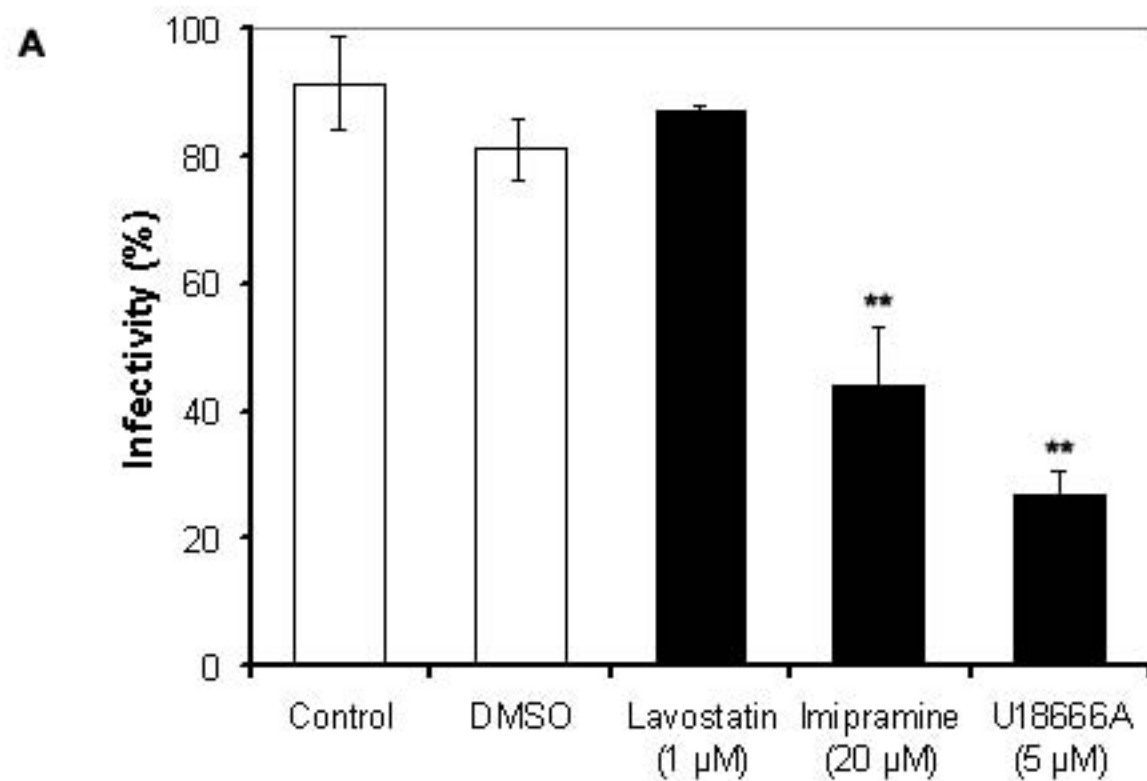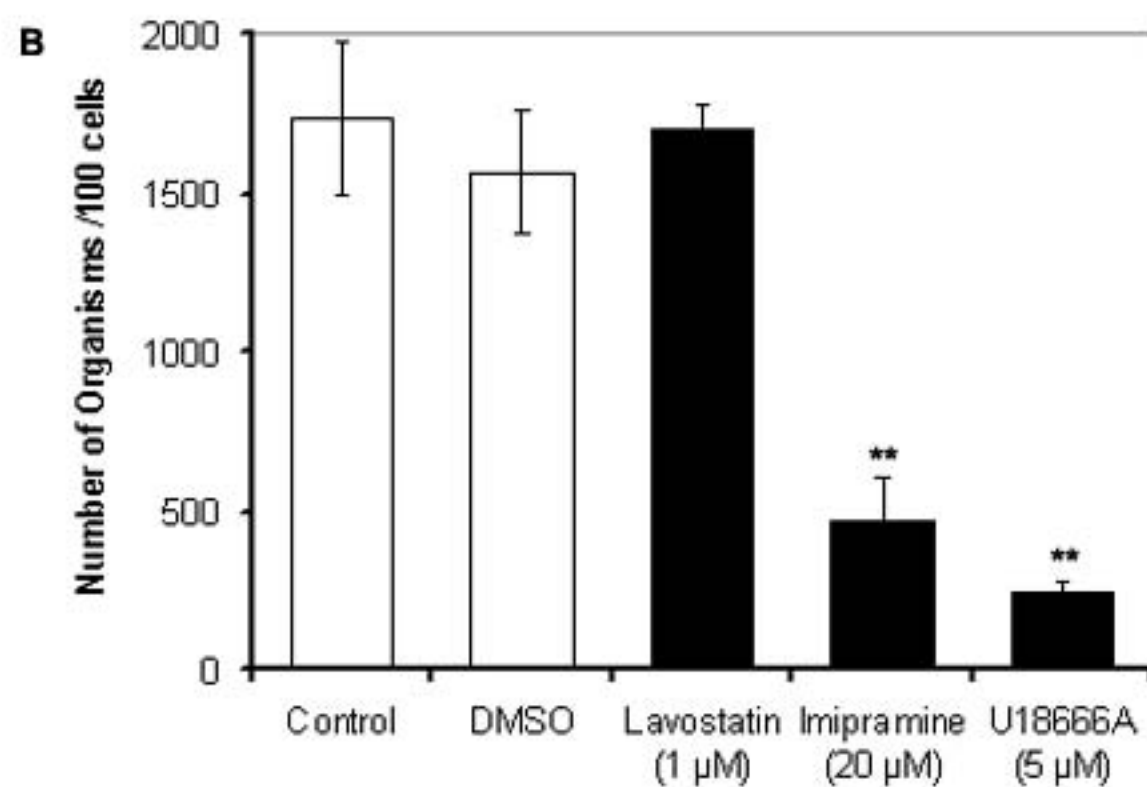

Supplement: Figure S1 — Cholesterol transport and biosynthesis inhibitors U18886A, imipramine, and lovastatin were added into RF/6A cells at 3 h p.i. at the indicated dosages, and infection percentage (A) and numbers of bacteria (B) were determined on day 3 p.i.. Data are expressed as mean±standard deviation (n = 3) and are representative of three independent experiments with similar results. **, p<0.01 (unpaired two-tailed t-test). (0.04 MB PDF) [file ppat.1000329.s001.pdf]
